# Synthesis of acridone derivatives via heterologous expression of a plant type III polyketide synthase in *Escherichia coli*

**DOI:** 10.1186/s12934-020-01331-2

**Published:** 2020-03-20

**Authors:** Gyu-Sik Choi, Hye Jeong Choo, Bong-Gyu Kim, Joong-Hoon Ahn

**Affiliations:** 1 Department of Bioscience and Biotechnology, Bio/Molecular Informatics Center, Konkuk University, Seoul, 05029 Republic of Korea; 2 Department of Forest Resources, Gyeongnam National University of Science and Technology, 33 Dongjin-ro, Jinju-si, Gyeongsangman-do 52725 Republic of Korea

**Keywords:** Acridone, Metabolic engineering, Polyketide synthase

## Abstract

**Background:**

Acridone alkaloids are heterocyclic compounds that exhibit a broad-range of pharmaceutical and chemotherapeutic activities, including anticancer, antiviral, anti-inflammatory, antimalarial, and antimicrobial effects. Certain plant species such as *Citrus microcarpa*, *Ruta graveolens*, and *Toddaliopsis bremekampii* synthesize acridone alkaloids from anthranilate and malonyl-CoA.

**Results:**

We synthesized two acridones in *Escherichia coli.* Acridone synthase (*ACS*) and anthraniloyl-CoA ligase genes were transformed into *E. coli*, and the synthesis of acridone was examined. To increase the levels of endogenous anthranilate, we tested several constructs expressing proteins involved in the shikimate pathway and selected the best construct. To boost the supply of malonyl-CoA, genes coding for acetyl-coenzyme A carboxylase (*ACC*) from *Photorhabdus luminescens* were overexpressed in *E. coli*. For the synthesis of 1,3-dihydroxy-10-methylacridone, we utilized an *N*-methyltransferase gene (*NMT*) to supply *N*-methylanthranilate and a new *N*-methylanthraniloyl-CoA ligase. After selecting the best combination of genes, approximately 17.3 mg/L of 1,3-dihydroxy-9(10H)-acridone (DHA) and 26.0 mg/L of 1,3-dihydroxy-10-methylacridone (NMA) were synthesized.

**Conclusions:**

Two bioactive acridone derivatives were synthesized by expressing type III plant polyketide synthases and other genes in *E. coli*, which increased the supplement of substrates. This study showed that is possible to synthesize diverse polyketides in *E. coli* using plant polyketide synthases.

## Background

Natural compounds are valuable in cosmetics, food, and pharmaceutical industries [1]. Therefore, natural and nature-inspired, chemically synthesized compounds have extensively been developed and exploited for countless industrial purposes. Phytochemicals are typical natural compounds that have additional biological, nutritive, and/or pharmacological value. Among the diverse phytochemicals, secondary metabolites such as alkaloids, phenylpropanoids, and terpenoids have been extensively studied, and some of them have been employed in various fields [2].

Acridones are heterocyclic alkaloids that contain a tricyclic ring with nitrogen at the 10th position and a carbonyl group at the 9th position [3]. Acridone alkaloids are secondary metabolites that are generally found in the plant family, Rutaceae [4]. Various acridone derivatives (glyforine, acronycine, thioacridones, and substituted 9-aminoacridines, etc.) have been reported to exert a wide range of chemotherapeutic effects including anticancer, antimicrobial, antimalarial, antipsoriatic activities [5–8]. The synthesis of acridone alkaloids in plants (Rutaceae family) was reported several decades after the discovery of acridine as a derivative of coal tar [9].

*N*-methylacridone (1,3-dihydroxy-10-methylacridone) was discovered in *Ruta graveolens* [10]. Some genes in acridone biosynthesis have been characterized, and the acridone biosynthetic pathway has been elucidated in *R. graveolens*. The first committed step in acridone alkaloid biosynthesis is the conversion of anthranilate into *N*-methylanthranilate by anthranilate *N*-methyltransferase (ANMT). Next is the *N*-methylanthranilate-catalyzed synthesis of *N*-methylanthraniloyl-CoA using coenzyme A (CoA). Then, acridone synthase (ACS)—one of the plant polyketide synthases (PKSs)—catalyzes the condensation of *N*-methylanthraniloyl-CoA and malonyl-CoA. *ANMT* and *ACS* were cloned in *R. graveolens* [11, 12]. *ACS*s from *Huperzia serrata* [13] and *Citrus microcarpa* [14] have also been cloned. Notably, to date, plant anthranilate coenzyme A ligase has not been cloned.

Microorganic biosynthetic platforms have emerged as the leading platforms for the production of natural and synthetic value-added compounds, such as flavonoids, alkaloids, polyketides, and various chemicals. Due to its well-established genetics and physiology, *Escherichia coli* has become one of the representative microorganisms in biosynthetic platforms [15]. One of the secondary metabolic pathways of *E. coli*, the shikimate pathway has received considerable attention as it is a major pathway for the production of aromatic compounds [16]. Biosynthetic pathways for aromatic amino acid production (l-tryptophan, l-tyrosine and l-phenylalanine) including the shikimic acid pathway provide the chemical building blocks for the synthesis of various chemicals through specific intermediates, such as chorismate and shikimate [17–21].

**Fig. 1:**
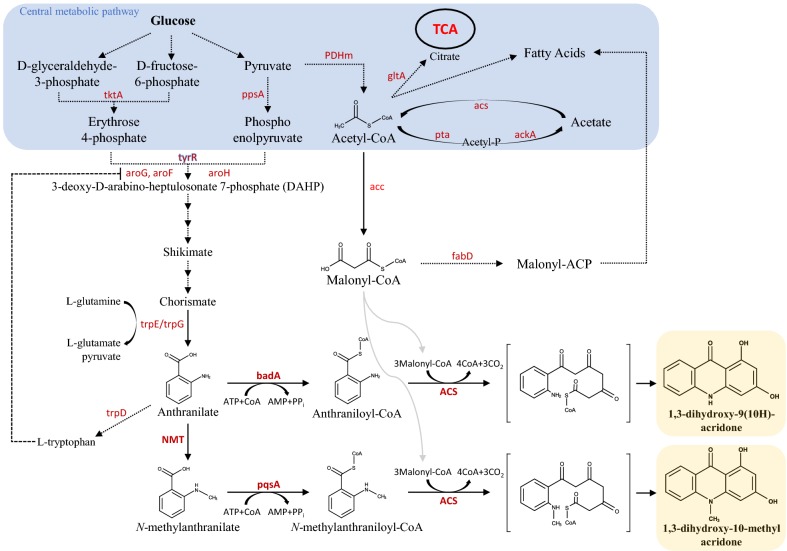
Biosynthesis scheme for producing two acridone derivatives in *Escherichia coli*. Dotted arrows represent the metabolic pathways in *E. coli*. Bold arrows indicate the pathway engineered for the synthesis of the two acridones (dihydroxyacridone (DHA) and *N*-methylacridone (NMA)). Introduced genes are as follows: ACS, acridone synthase from *Ruta graveolens*; badA, benzoate CoA ligase from *R. palustris*; pqsA, anthranilate coenzyme A ligase from *P. aeruginosa*; NMT, *N*-methyltransferase from *Ruta graveolens*; acs, acetyl-CoA synthase, PDH, pyruvate dehydrogenase complex, ackA & pta, acetate kinase A & phosphate acetyltransferase from *E. coli*; and acc, acetyl-coenzyme A carboxylase from *Photorhabdus luminescens*

We synthesized two acridones (1,3-dihydroxy-9(10*H*)-acridone [DHA] and 1,3-dihydroxy-10-methylacridone [NMA]) using engineered *E. coli* and two substrates, namely anthranilate, and malonyl-CoA. To optimize the substrate supply for the synthesis of acridone, we prepared several sets of constructs; the first set for the synthesis of anthranilate using genes coding for proteins involved in the shikimate pathway and the second set for the synthesis of malonyl-CoA by overexpressing acetyl-coenzyme A carboxylases (*ACCs*). For the synthesis of NMA (1,3-dihydroxy-10-methylacridone), we additionally introduced the *N*-methyltransferase gene (*NMT*) to supply *N*-methylanthranilate by using endogenous anthranilate. The overall scheme of the biosynthesis of these two compounds is shown in Fig. 1. Through a combination of these genes along with *ACS*, *badA*, and *pqsA*, which are involved in CoA utilization or substrate cyclization, we were able to synthesize 17.3 mg/L DHA and 26.0 mg/L NMA.

## Results

### Screening of constructs to synthesize DHA and NMA

**Fig. 2:**
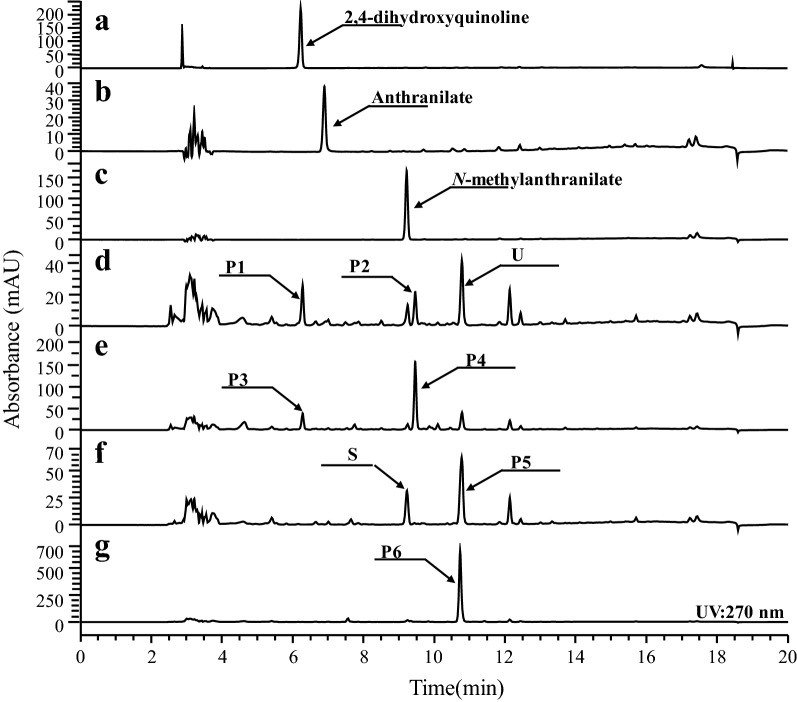
Screening of PKS and coenzyme ligase for the in vivo synthesis of DHA and NMA. **a** Authentic 2,3-dihydroxyquinoline (DHQ); **b** authentic anthranilate; **c** authenic *N*-methylanthranilate; **d** products from *E. coli* harboring pC-CmACS-BadA; **e** products from *E. coli* harboring pC-RgACS-BadA; **f** products from *E. coli* harboring pC-CmACS-pqsA; **g** products from *E. coli* harboring pC-RgACS-pqsA. *E. coli* harboring pC-CmACS-BadA (**d**), pC-RgACS-BadA (**e**), pC-CmACS-pqsA (**f**) or pC-RgACS-pqsA (**g**) were supplied with 100 μM anthranilate (**d**, **e**) or *N*-methylanthranilate (**f**, **g**). P1 and P3 were DHQ. P2 and P4 were determined to be DHA by NMR. S was unreacted *N*-methylanthranilate. P5 and P6 were determined to be NMA by NMR. U was unidentified product which seemed to be an intermediate of DHA and its retention time was slightly different from that of NMA

DHA and NMA are synthesized from anthranilate or *N*-methylanthranilate and malonyl-CoA, respectively. Anthranilate and *N*-methylanthranilate are activated by coenzyme A. We tested two CoA ligases, *badA*—encoding benzoate coenzyme A ligase—and *pqsA* encoding anthranilate coenzyme A ligase. Two ACSs, RgACS, and CmACS were tested. *E. coli*—harboring each of the four constructs pC-RgACS-badA, pC-CmACS-badA, RgACS-pqsA or pC-CmACS-pqsA—was exposed to 100 μM anthranilate or *N*-methylanthranilate. A new peak was observed in culture filtrates from *E. coli* strains harboring RgACS-badA or pC-CmACS-badA when they were supplied with anthranilate (Fig. 2d, e). The molecular mass of the synthesized product was 227.06 Da, which corresponded to the predicted mass of DHA. However, *E. coli* cells harboring RgACS-pqsA or pC-CmACS-pqsA that were supplied with *N*-methylantrhanilate synthesized a new product whose molecular mass was 240.87 Da, which is the predicted mass of NMA (Fig. 2e, g). Based on the structure—using nuclear magnetic resonance spectroscopy (NMR)—we confirmed that the two compounds were DHA and NMA, respectively, (see “Methods”). These results indicated that badA could potentially convert anthranilate into anthraniloyl-CoA and that pqsA is responsible for the conversion of *N*-methylanthranilate into *N*-methylanthraniloyl-CoA.

*Escherichia coli* strains harboring *RgACS* synthesized 11.80 mg/L DHA (51.96 μM) when 100 μM anthranilate was supplied, and synthesized 17.52 mg/L (72.62 μM) NMA when 100 μM *N*-methylanthranilate was provided. This yield exceeded that obtained using *E. coli* harboring *CmACS*, which synthesized 1.4 mg/L DHA and 6.0 mg/L NMA. In addition, the amount of byproduct such as 2,3-dihydroxyquinoline (DHQ) were found more in *E. coli* harboring *CmACS* and the unreacted *N*-methylanthranilate was observed in *E. coli* harboring *CmACS*. This result indicates that RgACS effectively synthesizes DHA and NMA. We observed the synthesis of 2,4-dihydroxyquinline (DHQ) in *E. coli* strains harboring RgACS-badA or CmACS-badA. DHQ also used anthranoyl-CoA and malonyl-CoA. Two molecules of malonyl-CoA instead of three, are used to synthesize DHQ. The amount of the synthesized DHQ was 2.6 mg/L in *E. coli* harboring CmACS-badA and 3.6 mg/L in *E. coli* harboring RgACS-badA, while the amount of DHA was 1.3 mg/L in CmACS and 10.5 mg/L in RgACS.

The synthesis of *N*-methylquinoline (NMQ) was observed in the culture filtrate of *E. coli* harboring CmACS-pqsA. Nevertheless, we could not observe any detectable NMQ in *E. coli* harboring RgACS-pqsA. Enzymatic reactions with *N*-methylanthranilate using CmANS revealed that the synthesized products resulted from the incorporation of two (*N*-methylquinolone) or three molecules (*N*-methylacridone) of malonyl-CoA with a preference towards *N*-methylacridone synthesis [14]. However, the enzymatic reaction using RgACS with *N*-methylanthranilate produced only NMA (but not *N*-methylquinolone) [22]. These results indicate that RgACS is better than CmACS at synthesizing DHA and NMA. Therefore, we selected constructs containing RgACS for further experiments.

### Synthesis of NMA

**Fig. 3:**
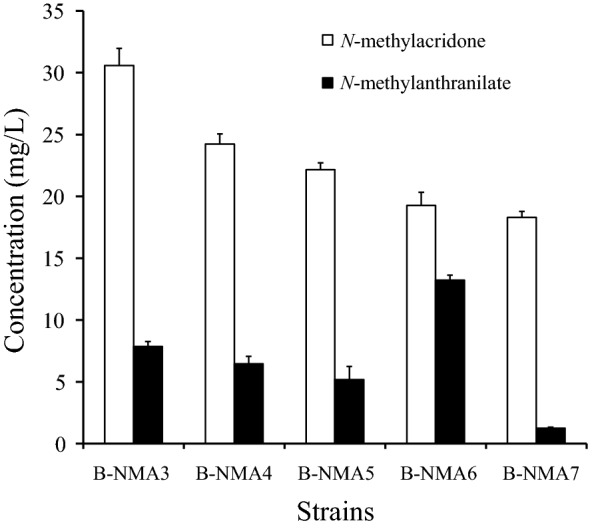
Effect of the precursor pathway genes on the production of *N*-methylacridone. B-NMA3 overexpressed *acc* (acetyl-CoA carboxylase), B-NMA4 overexpressed *ackA* (acetate kinase), and *pta* (phosphate acetyltransferase), B-NMA5 overexpressed *PDHm* (pyruvate dehydrogenase complex variant), and B-NMA6 overexpressed *acs* (acetyl-coenzyme A synthetase)

*N*-methylanthranilate is the building block of NMA, but *E. coli* does not synthesize *N*-methylanthranilate. Anthranilate NMT was employed to synthesize NMA. In order to increase the substrate for NMT, *trpE* was overexpressed. The second substrate of NMA synthesis is malonyl-CoA. The effects the four constructs that reportedly affect intracellular malonyl-CoA were individually tested with respect to NMA synthesis. Three of them (PDHm, acs, and ackA-pta) increased the level of acetyl-CoA [23, 24] and one of them (acc) synthesized malonyl-CoA from acetyl-CoA [24]. We engineered five *E. coli* strains (B-NMA3‒B-NMA7) and tested the synthesis of NMA. Four strains synthesized NMA. Among them, the strain B-NMA3 produced the highest amount of NMA (30.6 mg/L) followed by B-NMA4 (24.2 mg/L), B-NMA5 (22.2 mg/L), B-NMA6 (19.3 mg/L), and B-NMA7 (18.3 mg/L) (Fig. 3). The *E. coli* strains harboring pE-RgACS-PqsA, pC-NMT-TrpE, and the empty pA vector synthesized approximately 18.3 mg/L NMA. The overexpression of *acc* increased NMA synthesis (~ 1.7-fold), followed by *pta*-*ackA* (~ 1.3-fold), *PDHm* (~ 1.2-fold), and *acs* (~ 1.1-fold). These results indicated that the overexpression of gene involved in acetyl-CoA or malonyl-CoA increased the synthesis of NMA and the enhancement of malonyl-CoA synthesis by acc is more effective in the synthesis of NMA than the increase of acetyl-CoA by pta-ackA, PDHm, or acs.

We also tried to increase endogenous anthranilate levels by overexpressing *aroG* and the feedback-inhibition-free version of *aroG* (*aroG*^*f*^). Two more *E. coli* strains (B-NMA-8 and B-NMA-9) were tested. However, we could not detect the synthesis of NMA. Only the accumulation of anthranilate and *N*-methylanthranilate was observed. The unreacted anthranilate and *N*-methylanthranilate in B-NMA-8 were 16.0 and 35.0 mg/L, respectively; only 7.2 mg/L *N*-methylanthranilate was observed in B-NMA-3, whereas anthranilate was not observed. The rapid synthesis of anthranilate or *N*-methylanthranilate seemingly inhibited the synthesis of NMA. Notably, higher copy number plasmids containing RgACS and pqsA did not further increase NMA synthesis. Likely, the activities of these two downstream proteins got saturated when converting the synthesized *N*-methylanthranilate into NMA. Fine-tuning of the whole process is critical to increasing the final yield of the product [25, 26].

**Fig. 4:**
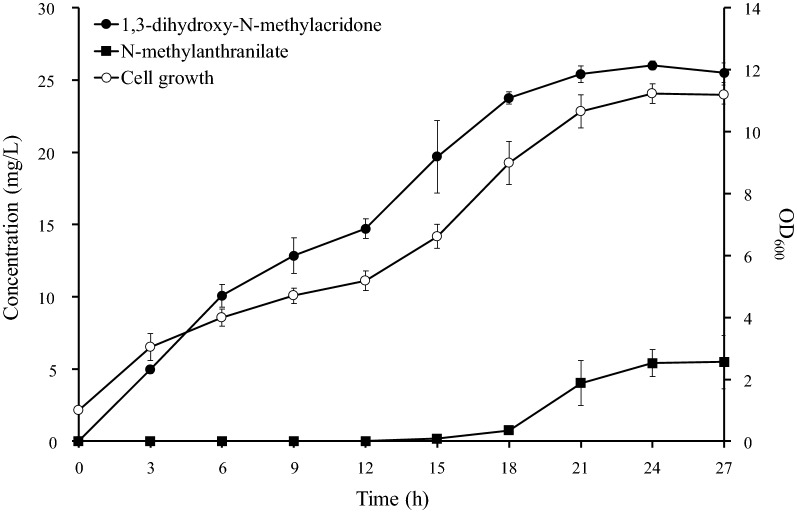
Production of *N*-methylacridone using the strain B-NMA3

Using the strain B-NMA3, we monitored the synthesis of NMA and *N*-methylanthranilate for 27 h. The synthesis of both NMA and *N*-methylanthranilate showed a similar pattern (Fig. 4). Both reached their maximal synthesis at 24 h, at which time, approximately 26.0 mg/L NMA and 5.4 mg/L *N*-methylanthranilate were synthesized.

### Synthesis of DHA

**Fig. 5:**
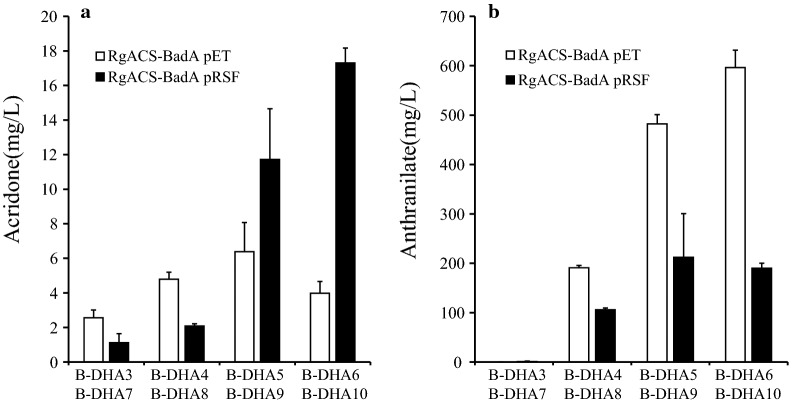
Optimization of the precursor and vector copy number for acridone production. **a** Production of acridone; **b** production of anthranilate

Anthranilate and malonyl-CoA are substrates for DHA. Endogenous levels of these two compounds are probably critical determinants of the final yield. To increase DHA synthesis, we used two strategies. The first strategy was to increase endogenous anthranilate. The shikimate pathway synthesizes anthranilate. Genes in this pathway were overexpressed. The second strategy was to use a plasmid with different copy number to express *RgACS* and *badA*. We constructed eight different *E. coli* strains. The levels of synthesized DHA increased from 2.56 in B-DHA3 to 6.39 mg/L in D-BHA5, and the strain D-BHA6 produced approximately 3.98 mg/L of DHA. Importantly, the levels of unreacted anthranilate continued to increase, and were 0.72 mg/L in B-DHA3 and 593.40 mg/L in B-DHA6. It seemed that higher production of anthranilate inhibited the synthesis of DHA, and that the conversion of the synthesized anthranilate into DHA was critical for increasing the yield of DHA. In order to augment the conversion of anthranilate, greater and better involvement of downstream genes (*badA* and *RgACS*) seems necessary. Therefore, we tested the strain B-DHA7-10. The synthesis of DHA increased from 1.12 mg/L in B-DHA7 to 17.3 mg/L in B-DHA10 (Fig. 5). In particular, the strain(s) that were expected to synthesize more anthranilate produced more DHA. Besides, the levels of unreacted anthranilate in these strains were less than those in corresponding strains harboring a lower copy of *badA* and *RgACS*. Taken together, the higher copy number of *RgACS* and *badA* facilitated the synthesis of DHA.

**Fig. 6:**
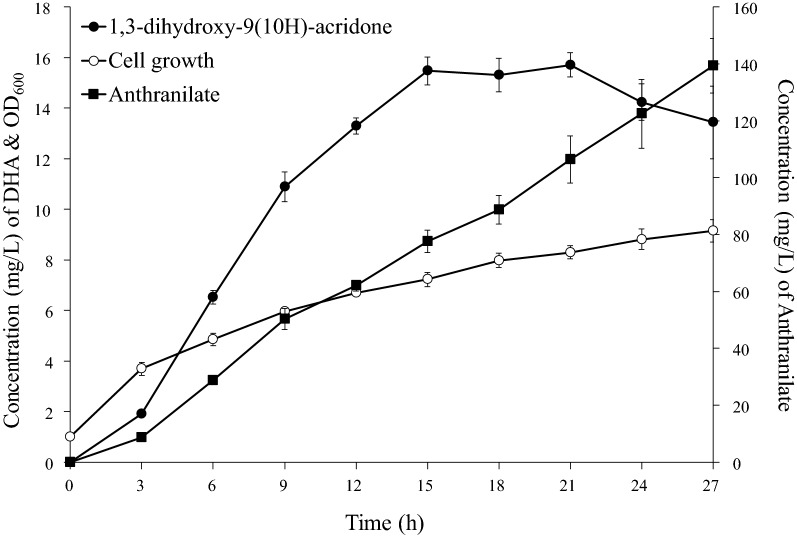
Production of dihydroxyacridone using the strain B-DHA10

We also tested the four constructs that were supposed to increase intracellular levels of malonyl-CoA. Like for the synthesis of NMA, the placcABCD was also the best in the context of DHA synthesis (data not shown). The synthesis of DHA in the strain B-DHA10 was monitored (13 mL of 1% YM9 broth in 100 mL flask). DHA levels continued to increase until 15 h and remained almost the same until 21 h. The levels of unreacted anthranilate also continued to increase until 40 h. Approximately 15.7 mg/L DHA was synthesized at 24 h (Fig. 6).

## Discussion

In our present works, we successfully synthesized two acridone derivatives, 1,3-dihydroxy-9(10*H*)-acridone and 1,3-dihydroxy-10-methylacridone, using engineered *E. coli*. Genes coding for proteins in the shikimate pathway and *TrpE* encoding anthranilate synthase were tested and selected for the synthesis of the 1st substrate, anthranilate. Acetyl-CoA-carboxylase from *P. luminescens* was introduced to increase the available levels of the 2nd substrate, malonyl-CoA (for ACS). We tested ACS from *R. graveolens* and *C. microcarpa* to select the one that was better with respect to the synthesis of DHA and NMA. The results of in vitro enzymatic efficacy tests showed that ACS from *R. graveolens* outperformed that from *C. microcarpa* [11, 14, 22]. Sometimes, in vitro enzymatic results did not correlate with the in vivo results due to the presence of unknown substrates in vivo, which expectedly inhibit or divert the enzymatic activity [27]. Therefore, we tested the in vivo synthesis of acridone using both genes. In this study, in vivo biosynthesis of acridones by RgACS showed better productivity than CmACS. Based on the in vitro properties of ACS and on the in vivo acridone biosynthesis experiment, we could identify a positive correlation between enzyme properties and acridone biosynthesis.

In order to increase the final yield of the two acridones, we tested the genes coding for proteins involved in the shikimate pathway. We observed a dramatic increase in the levels of intermediates, such as anthranilate, instead of an increase in DHA levels during DHA synthesis. Importantly, during the synthesis of DHA, the rate-limiting step was likely the conversion of anthraniloyl-CoA into DHA by PKS. However, the conversion of anthranilate into *N*-methylanthranilate and/or the conversion of *N*-methylanthraniloyl-CoA into NMA were limiting steps during the synthesis of NMA. Exposure of *E. coli* harboring CoA ligase and PKS to either anthranilate or *N*-methylanthranilate resulted in no further synthesis of DHA and NMA, (~ 500 μM of anthranilate and 300 μM of *N*-methylanthranilate). The endogenous levels of anthranilate upon expressing the genes coding for proteins in the shikimate pathway increased more than 500 μM (Fig. 5b), a concentration at which the synthesis of DHA is likely inhibited. These findings indicated that PKS was probably a rate-limiting step. In case of NMA synthesis, we found that the conversion of anthranilate into *N*-methylanthranilate was a limiting step [21]. In vitro enzymatic study using the purified ACS from *R. graveolens* also showed that ACS was inhibited by 250 μM *N*-methylanthraniloyl-CoA [28] The construct that minimizes the accumulation of the anthranilate appeared to be the best for the synthesis of NMA and DHA. Fine-tuning of the overall pathway is critical to enhancing the final yield of the product.

Aerobic growth of *E. coli* produces ATP, ubiquinol-8, CO_2_ and a considerable amount of acetic acid as a byproduct through the acetate producing pathways [29]. The production of acetic acid could be a negative influence on the synthesis of acridone derivatives. The synthesized acetic acid is neutralized upon converting acetic acid into acetyl-CoA by acetyl-CoA synthase (acs) [30]. Acetyl-CoA is then converted into malonyl-CoA by ACC. Overexpression of *acs* or *acc* enhanced the production of DHA and NMA because it resulted not only in the supply of the second substrate (malonyl-CoA), but also in the reduction of the byproduct, acetic acid. These results agreed with the previous reports that overexpression of *acs*, *acc*, or *PDH* enhanced the synthesis of flavonoids and triacetic acid lactone [23, 24, 31].

## Conclusions

We synthesized two acridones (DHA and NMA) in *Escherichia coli* using two substrates, namely anthranilate and malonyl-CoA. Towards this, plant acridone synthase (*ACS*) and anthraniloyl-CoA ligase genes were transformed into *E. coli*. To optimize the substrate supply for acridone synthesis, we prepared several sets of constructs; the first set for the synthesis of anthranilate using genes coding for proteins involved in the shikimate pathway—major pathway for the production of aromatic compounds—and the second set for the synthesis of malonyl-CoA by overexpressing acetyl-coenzyme A carboxylases (*ACCs*). For the synthesis of NMA, we additionally introduced the *N*-methyltransferase gene (*NMT*) to supply *N*-methylanthranilate by using endogenous anthranilate. Through a combination of these genes along with *ACS*, *badA* and *pqsA*, which are involved in CoA utilization or substrate cyclization, we were able to synthesize 17.3 mg/L DHA and 26.0 mg/L NMA.

## Methods

### Constructs

*ACS* from *R. graveolens* (RgACS; GenBank: AJ297786.2) was cloned using reverse transcription and polymerase chain reaction (RT-PCR). Two primers 5′-ATGGATCCGATGGAATCCCTGAAGGAGATG-3′ and 5′-ATGCGGCCGCTCATGCTTCAACGGGGACAC-3′ were used (restriction sites for *Eco*RI and *Not*I have been underlined). ACS from *Citrus microcarpa* (GenBank: AB823699) was also cloned by RT-PCR using two primers 5′-aagaattcaATGGTAACCATGGAGGAGATTA-3′ and 5′-aagcggccgcTCATGCTTCTATAGGGAGACTGTG-3′ (restriction sites for *Eco*RI and *Not*I have been underlined).

*badA* from *Rhodopseudomonas palustris* (GenBank: L42322.1) and *pqsA* from *Pseudomonas aeruginosa* (*Nde*I/*Xho*I) had been previously cloned 21. *badA* was first cloned into pCDF-duet1 (*Nde*I/*Kpn*I) and then *ACS* was subcloned into pCDF-duet1 containing badA (pC-RgACS-badA) (*Bam*HI/*Not*I). Subsequently, RgACS-badA was subcloned into pET-duet1 and pRSF-duet1 (*Bam*HI/*Eco*RV) using polymerase chain reaction (PCR). Two primers 5′-ATGGATCCGATGGAATCCCTGAAGGAGATG-3′ and 5′-GCGGCCGCTCAGCCCAACACACCCTCG-3′ were used (restriction sites for *Bam*HI and *Not*I have been underlined). *pqsA* was first cloned into pCDF-duet1 (*Nde*I/*Xho*I) and ACS was subcloned into pCDF-duet1 containing pqsA (pC-RgACS-pqsA) (*Bam*HI/*Not*I) and then RgACS-pqsA was subcloned into pET-duet1 (pE-RgACS-pqsA) (*Bam*HI/*Xho*I).

pA-accABCD encoding acetyl-CoA carboxylase had been cloned previously [32]. *TrpE* was cloned as described in Lee et al. [20] and subcloned into pCDF-duet1 (pC-trpE) (*Bam*HI:*Bgl*II/*Xho*I). aroG and aroG^f^—both of which had been previously cloned [18]—were subcloned into pC-trpE (*Eco*RI/*Not*I). pC-aroL-aroE-aroD-aroB-aroG^f^-ppsA-tktA-trpE had been constructed previously [21]. The *E. coli* pyruvate dehydrogenase complex variant (PDHm) was cloned as described in Bocanegra et al. [33]. *ackA* encoding acetate kinase (AAC75356) and *pta* encoding phosphate acetyltransferase (AAC75357) were cloned from *E. coli* using PCR. *pta* was subcloned into pACYC-duetI (*Nde*I/*Xho*I), and ackA was subcloned into the resulting plasmid (*Eco*RI/*Hind*III) to give rise to pA-ack-pta. *acs*—encoding *E. coli* acetyl-coenzyme A synthetase (BAE78071)—was subcloned into pACYC-duetI (*Eco*RI/*Hind*III) (pA-acs). *ACC* encoding acetyl-CoA carboxylase from *Photorhabdus luminescens* (*accABCD*) had been cloned previously [21].

NMT from *Ruta graveolens* had been cloned previously [20]. In order to prepare the pC-aroG-NMT-trpE construct, *trpE* was amplified using a forward primer containing a *Bam*HI site and a reverse primer containing a *Xho*I site, and was subcloned into pCDF-duet1 (*Bgl*II/*Bam*HI) (pC-trpE). *NMT* was amplified with a forward primer containing a *Bam*HI site, and a reverse primer containing an *Afl*II site, following which it was subcloned into pC-trpE (*Bam*HI/*Afl*II). The resulting construct was digested with *Bam*HI/*Xho*I, and was then subcloned into pCDF-duet1 (*Bgl*II/*Xho*I) (pC-NMT-trpE). aroG or aroG^f^ were amplified using primers containing *Eco*RI (forward primer) and *Not*I sites (reverse primer) and were subcloned into pC-NMT-trpE (*Eco*RI/*Not*I). The constructs and the strains used in this study were listed in Table 1.

**Table 1:** Plasmids and strains used in the present study

Plasmids or *E. coli* strain	Relevant properties or genetic marker	Source or reference
Plasmids		
pACYCDuet-1	P15A ori, Cm^r^	Novagen
pCDFDuet-1	CloDE13 ori, Str^r^	Novagen
pETDuet-1	f1 ori, Amp^r^	Novagen
pRSFDuet-1	RSF ori, Kana^r^	Novagen
pC-CmACS-badA	pCDFDuet + *ACS* from *Citrus microcarpa *+* badA* from *Rhodopseudomonas palustris*	This study
pC-RgACS-badA	pCDFDuet + *ACS* from *Ruta graveolens* +* badA* from *R. palustris*	This study
pC-RgACS-pqsA	pCDFDuet + *ACS* from *Ruta graveolens* +* pqsA* from *Pseudomonas aeruginosa*	This study
pC-CmACS-pqsA	pCDFDuet + *ACS* from *C. microcarpa* + *pqsA* from *P. aeruginosa*	This study
pE-RgACS-badA	pETDuet + *ACS* from *Ruta graveolens* +* badA* from *R. palustris*	This study
pE-RgACS-pqsA	pETDuet + *ACS* from *Ruta graveolens* +* pqsA* from *P. aeruginosa*	This study
pR-RgACS-badA	pRSFDuet + *ACS* from *Ruta graveolens* +* badA* from *R. palustris*	This study
pA-accABCD	pACYCDuet + *accABCD* from *Photorhabdus luminescens*	Kim et al. [32]
pA-Acs	pACYCDuet + *Acs* from *Escherichia coli*	This study
pA-ackA-pta	pACYCDuet + *ackA*-*pta* from *E. coli*	This study
pA-PDHm	pACYCDuet + *PDHm* from *E. coli*	This study
pC-trpE	pCDFDuet + *trpE* from *E. coli*	This study
pC-aroG-trpE	pCDFDuet + *aroG* from *Escherichia coli* in the first multiple cloning site (MCS1) + *trpE* from *E. coli* in the second MCS (MCS2)	This study
pC-aroG^f^-trpE	pCDFDuet + *aroG*^*f*^ from *E. coli* in MCS1 + *trpE* in MCS2	This study
pC-aroG^f^-ppsA-tktA-trpE	pCDFDuet + *aroG*^*f*^, *ppsA* and *tktA* from *E. coli* in MCS1 + *trpE* in MCS2	This study
pC-aroL-aroG^f^-ppsA-tktA-trpE	pCDFDuet + *aroL*, *aroG*^*f*^, *ppsA* and *tktA* from *E. coli* in MCS1 + *trpE* in MCS2	This study
pC-aroL-aroE-aroD-aroB-aroG^f^-ppsA-tktA-trpE	pCDFDuet + *aroL*, *aroE*, *aroD*, *aroB*, *aroG*^*f*^, *ppsA* and *tktA* from *E. coli* in MCS1 + *trpE* in MCS2	This study
pC-NMT	pCDFDuet + *NMT* from *Ruta graveolens*	This study
pC-NMT-trpE	pCDFDuet + *NMT* from *R. gravealens* +* trpE* from *E. coli*	This study
pC-aroG-NMT-trpE	pCDFDuet + *aroG* from *E. coli* in MCS1 + *NMT* and *trpE* in MCS2	This study
pC-aroG^f^-NMT-trpE	pCDFDuet + *aroG*^*f*^ from *E. coli* in MCS1 + *NMT* and *trpE* in MCS2	This study
Strains		
DH5α	F– φ80lacZΔM15 Δ(lacZYA-argF)U169 recA1 endA1 hsdR17(rK–, mK +) phoA supE44 λ− thi-1 gyrA96 relA1	Novagen
BL21 (DE3)	F^−^*ompT hsdS*_*B*_(r_B_^−^ m_B_^−^) *gal dcm lon* (DE3)	Novagen
B-DHA1	*E. coli* BL21 (DE3) harboring pC-RgACS-BadA	This study
B-DHA2	*E. coli* BL21 (DE3) harboring pC-CmACS-BadA	This study
B-DHA3	*E. coli* BL21 (DE3) harboring pE-RgACS-BadA, pA-PlaccABCD, pC-TrpE	This study
B-DHA4	*E. coli* BL21 (DE3) harboring pE-RgACS-BadA, pA-PlaccABCD, pC-aroG-TrpE	This study
B-DHA5	*E. coli* BL21 (DE3) harboring pE-RgACS-BadA, pA-PlaccABCD, pC-aroG^f^-TrpE	This study
B-DHA6	*E. coli* BL21 (DE3) harboring pE-RgACS-BadA, pA-PlaccABCD, pC-aroL-aroE-aroD-aroB-aroG^f^-ppsA-tktA-trpE	This study
B-DHA7	*E. coli* BL21 (DE3) harboring pR-RgACS-BadA, pA-PlaccABCD, pC-TrpE	This study
B-DHA8	*E. coli* BL21 (DE3) harboring pR-RgACS-BadA, pA-PlaccABCD, pC-aroG-trpE	This study
B-DHA9	*E. coli* BL21 (DE3) harboring pR-RgACS-BadA, pA-PlaccABCD, pC-aroG^f^-trpE	This study
B-DHA10	*E. coli* BL21 (DE3) harboring pR-RgACS-BadA, pA-PlaccABCD, pC-aroL-aroE-aroD-aroB-aroG^f^-ppsA-tktA-trpE	This study
B-NMA1	*E. coli* BL21 (DE3) harboring pC-RgACS-PqsA	This study
B-NMA2	*E. coli* BL21 (DE3) harboring pC-CmACS-PqsA	This study
B-NMA3	*E. coli* BL21 (DE3) harboring pE-RgACS-PqsA, pC-NMT-TrpE, pA-PlaccABCD	This study
B-NMA4	*E. coli* BL21 (DE3) harboring pE-RgACS-PqsA, pC-NMT-TrpE, pA-ack-pta	This study
B-NMA5	*E. coli* BL21 (DE3) harboring pE-RgACS-PqsA, pC-NMT-TrpE, pA-mPDH	This study
B-NMA6	*E. coli* BL21 (DE3) harboring pE-RgACS-PqsA, pC-NMT-TrpE, pA-acs	This study
B-NMA7	*E. coli* BL21 (DE3) harboring pE-RgACS-PqsA, pC-NMT-TrpE, pACYCD	This study
B-NMA8	*E. coli* BL21 (DE3) harboring pE-RgACS-PqsA, pC-aroG-NMT-TrpE, pA-PlaccABCD	This study
B-NMA9	*E. coli* BL21 (DE3) harboring pE-RgACS-PqsA, pC-aorG^f^-NMT-TrpE, pA-PlaccABCD	This study

### Production and analysis of DHA and NMA in *E. coli*

The overnight cultures of *E. coli* transformants were inoculated into a fresh LB containing appropriate antibiotics and growth at 37 °C until OD_600_ = 1. Cells were harvested and resuspended in M9 medium containing 2% glucose, 1% yeast extract, antibiotics, 1 mM isopropyl β-d-1-thiogalactopyranoside (IPTG) in a test tube except that the synthesis of NMA and DHA was monitored for 27 h in a flask. The cells were grown at 30 °C with shaking for 24 h. The culture supernatant was extracted with three volumes of ethyl acetate (EA). The upper layer—after centrifugation—was collected and dried. The dried sample was dissolved in 60 μL dimethyl sulfoxide (DMSO).

To analyze the formation of DHA and NMA, Thermos Ultimate 3000 HPLC (high performance liquid chromatography) equipped with a photodiode array (PDA) detector and a Varian C18 reversed-phase column (Varian, 4.60 × 250 mm, 3.5 μm particle size) was used [21].

The synthesized DHA was purified using HPLC. The mobile phase consisted of water and acetonitrile (7:3, v/v), and no gradient was applied. The structure of the purified compounds was determined using proton nuclear resonance spectroscopy (NMR). DHA (1,3-dihydroxy-9(10H)-acridone), ^1^H NMR (500 MHz, DMSO-*d*_*6*_): δ 6.00 (d, *J* = 2.0 Hz, H-2), 6.30 (d, *J* = 2.0 Hz, H-4), 7.25 (ddd, *J* = 8.3, 7.0, 1.0 Hz, H-7), 7.47 (dd, *J* = 8.3, 1.4 Hz, H-8), 7.72 (ddd, *J* = 8.2, 7.0, 1.4, H-6), 8.15 (dd, *J* = 8.2, 1.0 Hz, H-5). To determine the structure of NMA (1,3-dihydroxy-10-methylacridone), thin layer chromatography (TLC, silica gel 60 F254, Millipore) was used to purify the putative NMA. Ethyl acetate and hexane (2:1 (v/v)) were used as developing solvents. The purified sample was dissolved in acetone-*d*_*6*_. The chemical shifts for ^1^H and ^13^C NMR data were referenced to that of tetramethylsilane (TMS). In order to verify the structure, COSY, TOCSY, NOESY, ^1^H-^13^C HMQC, and ^1^H-^13^C HMBC were used. The mixing time for TOCSY and NOESY was 60 ms and 1 s, respectively. The delay in the evolution of long-ranged couplings was 70 ms in HMBC. ^1^H NMR (500 MHz, acetone-*d*_*6*_): δ 6.19 (d, *J* = 1.9 Hz, H-2), 6.50 (d, *J *= 1.9 Hz, H-4), 7.78 (dd, *J* = 8.8, 1.2 Hz, H-5), 7.79 (m, H-6), 7.34 (m, H-7), 8.40 (dd, *J *= 8.0, 1.4 Hz, H-8), 3.90 (s, N-CH_3_). ^13^C NMR(500 MHz, acetone-*d*_*6*_): δ 95.26(C-2), 90.23(C-4), 114.7(C-5), 133.4(C-6), 120.6(C-7), 125.4(C-8), 179.9(C-9), 33.03(N-CH_3_). In the ^1^H spectrum, six peaks were observed in the aromatic region, while a single peak was observed at 3.90 ppm. All peaks in the aromatic region were assigned using COSY and TOCSY. The *N*-attached methyl group was thought to be responsible for the peak at 3.899 ppm as it showed two cross-peaks with H-5 and H-4 in NOESY.

## Data Availability

All data generated during this study are included in this published article.

## Abbreviations

ACC: Acetyl-coenzyme A carboxylase
ackA: Acetate kinase
ANMT: Anthranilate *N*-methyltransferase
ASC: Acridone synthase
CoA: Coenzyme A
DHA: 1,3-Dihydroxy-9(10*H*)-acridone
DMSO: Dimethyl sulfoxide
HPLC: High performance liquid chromatography
IPTG: Isopropyl β-d-1-thiogalactopyranoside
NMA: 1,3-Dihydroxy-10-methylacridone
NMR: Nuclear magnetic resonance spectroscopy
NMT: *N*-methyltransferase gene
PDA: Photodiode array
PDHm: Pyruvate dehydrogenase complex variant
PKS: Polyketide synthases
pta: Phosphate acetyltransferase
